# Testing a Flexible Method to Reduce False Monsoon Onsets

**DOI:** 10.1371/journal.pone.0104386

**Published:** 2014-08-08

**Authors:** Mathew Alexander Stiller-Reeve, Thomas Spengler, Pao-Shin Chu

**Affiliations:** 1 Uni Research Climate, Bergen, Norway; 2 Bjerknes Centre for Climate Research, Bergen, Norway; 3 Geophysical Institute, University of Bergen, Norway; 4 Department of Meteorology, University of Hawai'i, Manoa, Honolulu, United States of America; Institute of Tibetan Plateau Research, China

## Abstract

To generate information about the monsoon onset and withdrawal we have to choose a monsoon definition and apply it to data. One problem that arises is that false monsoon onsets can hamper our analysis, which is often alleviated by smoothing the data in time or space. Another problem is that local communities or stakeholder groups may define the monsoon differently. We therefore aim to develop a technique that reduces false onsets for high-resolution gridded data, while also being flexible for different requirements that can be tailored to particular end-users. In this study, we explain how we developed our technique and demonstrate how it successfully reduces false onsets and withdrawals. The presented results yield improved information about the monsoon length and its interannual variability. Due to this improvement, we are able to extract information from higher resolution data sets. This implies that we can potentially get a more detailed picture of local climate variations that can be used in more local climate application projects such as community-based adaptations.

## Introduction

In this paper, we develop and test a novel method for identifying monsoonal onsets and withdrawals in high-resolution gridded data. The novelty of our method lies in our definition of the transition between non-monsoon and monsoon seasons with the aim to reduce the need for spatial and temporal smoothing of the data, or for manual corrections of the results. Thus, we argue that our method can be used to extract time series from individual grid points or small regions to give accurate information of local climates. This more local information can thereafter be applied to local-scale climate applications.

Community based adaptation projects provide good examples of local-scale strategies aimed at individuals, families, villages, or districts (e.g. [1]). For such projects, we ideally need reliable information about past, present and future *local* climate [2]. In monsoon regions, one particular interest lies in the onset and withdrawal date of the monsoon, and in how much these dates vary interannually. After all, the timing of the monsoon onset is extremely significant, as ays can have large negative economical consequences especially for the poorest farming communities [3], [4]. The farmers in these communities are interested in information about the monsoon onset at their locality. For example, different agricultural communities in Bangladesh define the monsoon and it's onset in different ways [5]. Even though the monsoon is often considered as an essentially large-scale phenomenon, small regional-scale variations are clear.

**Figure 1:**
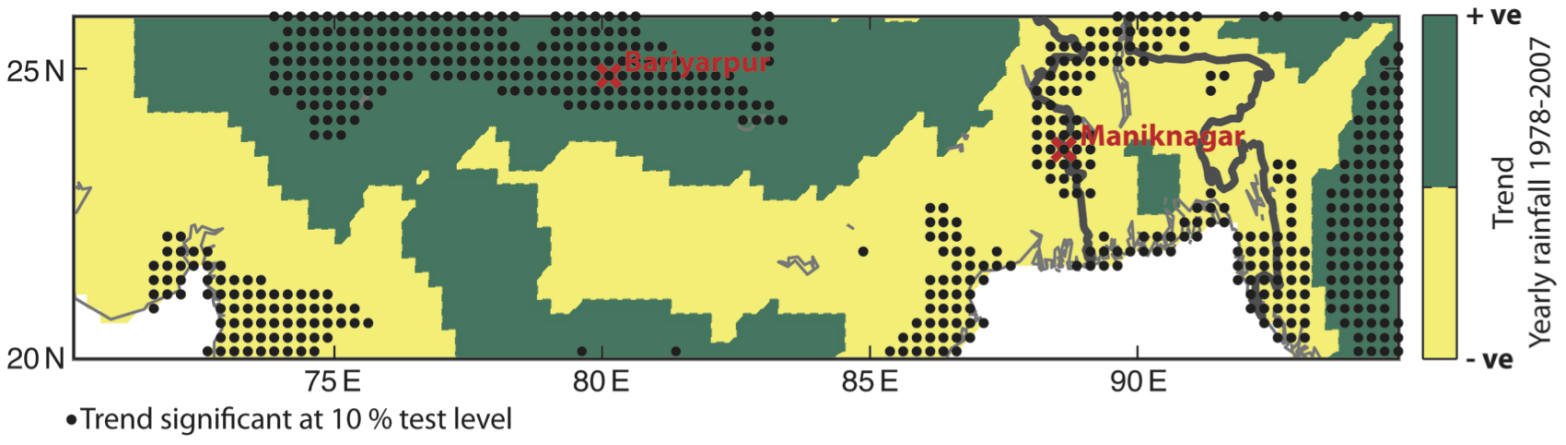
The trend in total yearly rainfall between 1978–2007 using the APHRODITE rainfall data set. The purpose of the figure is solely to illustrate the complexity in the monsoon system. For example, Bariyarpur experiences a negative trend of −14.7 mm mm/year/year, whereas Maniknagar has experienced a positive trend of +16.7 mm mm/year/year.

We illustrate these spatial variations by looking at the trends in total yearly rainfall within a single monsoon region between 1978–2007 as shown in Figure 1. We calculated the trends from the Asian Precipitation - Highly-Resolved Observational Data Integration Towards Evaluation of Water Resources (APHRODITE, V1101R1) rainfall data set [6], [7]. We calculated statistical significance using a bootstrapping method. We rejected our null hypothesis that the trend was non-zero if the trend lay outside the 5% and 95% percentiles of trends taken from 1000 random permutations of the time series at each grid point. The average trend over the whole region is +1.47 mm/year/year, which is not statistically significant. However, if we focus on local climate, then trends can become more significant. For example, in Bariyarpur, Madhya Pradesh, India, the trend was −14.7 mm/year/year, at the 10% significance test level. In Maniknagar, Bangladesh, the trend was +16.7 mm/year/year, at the 10% significance test level. This shows that we cannot apply information about the large-scale trend at locations where the local climate behaves very differently. We clearly need a local focus, if we aim to produce climate information that is useable [8] by people in places like Bariyarpur and Maniknagar, regional planners or other end users.

However, thinking local is fraught with challenges. In the absence of local meteorological observations, we ideally need high-resolution gridded numerical or reanalysis data to analyze local monsoon climates and related driving processes. There are several ongoing projects, which will provide high-resolution climate simulations and projections [9]. We face a well-known complication if we want to extract information about local monsoon onsets/withdrawals from such data sets, then. Previously we have often been unable to extract time series from grid points, due to the issue of false onsets [10], [11] Periods of unseasonal heavy rain or high convective activity can also cause false or bogus onsets, which usually happen long before the actual monsoon or rainy season starts. These false onsets obviously influence the analysis results.

If we include false onsets in time series, we artificially inflate the values of interannual variability. Previously, researchers have applied artificial cut-off dates [12] or smoothing techniques to reduce the effects of false onsets. For example, Zeng and Lu [13] declare the monsoon onset at a grid point only when the chosen criterion is exceeded at seven of the nine points centred at the grid point in question. Since they used 1×1 degree data, their approach yields representative results for the large-scale conditions, but not the local-scale, as all local meteorological processes are smoothed out. We emphasize that we are not criticizing previous methodologies. Previous studies have mostly been based on a basic research framework and have undoubtedly advanced our knowledge of the large-scale monsoon transitions. In a more applied research framework, we want to avoid smoothing so that we can create useable information about monsoon transitions at local scales. This information should be more easily applied to local decisions making processes. To achieve this, we have to reduce the occurrence of false onsets at the grid point scale!

With the aim of reducing the occurrence of false onsets in a high resolution data set, we extend the number of pentads (5-day periods) used in a monsoon definition to six as opposed to three or four that have previously been used [14], [15]. We will show that through the integration of background knowledge and a manual testing phase, our approach reduces the occurrence of false onsets. Because we integrate this background knowledge into the definition through a test phase, we call the method the Integrated Approach (IA). If the IA method reduces the occurrence of false onsets, we therefore reduce the need for spatial and temporal smoothing. In this case, we should be able to extract time series from individual grid points. This means that we will be able to use high-resolution information about the monsoon transitions in local applications.

In the next section we develop the IA method and explain how the algorithm identifies monsoonal onsets and withdrawals. We will also explain how we integrate background knowledge into the process. We complete the section by presenting a test case and the data. In section 3, we analyze the results from the test case. We analyse the results from separate grid points and a larger region and investigate how the IA affects the results of interannual variability. This indicates how successful the method is in reducing false onsets in the data. We conclude in the section 4 and make connotations about how we can apply this process to wider applications in climate services and adaptation strategies.

## Method

### 1. Background

Usually, when we define the monsoon onset/withdrawal, we firstly choose one or more meteorological parameters. A multitude of publications have defined the monsoon in various regions according to different climatic parameters, such as wind, rainfall, outgoing long wave radiation and atmospheric water content [12]–[20]. Secondly, we apply a criterion or threshold to the chosen parameter(s). The criterion identifies when a location –or effectively a grid point- experiences monsoon-like conditions. Thirdly, we allocate a seasonal transition to identify exactly when the monsoon season starts and ends. For example, if three consecutive pentad (5-day period) values of the specified parameter exceed the chosen threshold, then a monsoon transition is declared [14], [15], [21], [22]. Even though this process is usually applied in a basic research setting, it has potential in applied research or a climate application framework.

### 2. Algorithm

For our Integrated Approach (IA), we assume a monsoon parameter and threshold has been previously chosen in accordance with the requirements of the end-user or research aims. For example, the parameter could be rainfall with a threshold pentad value of 5 mm/day [19], or outgoing longwave radiation with a threshold pentad value of below 240 W/m^2^ [23] or 180 W/m^2^ [24]. Firstly, the algorithm tests each grid point time series separately and converts the pentad time series into a binary vector for each year separately, where 1 signifies a ‘hit’ -threshold exceeded- and 0 signifies a ‘miss’ -threshold not exceeded-. Thus, the result is a binary vector of 73 Julian pentads (JP) in a single year, e.g.,
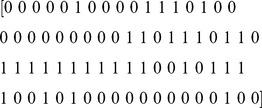
(2.1)


Next, a 6-pentad moving window from the binary vector is tested chronologically against each of the binary hit-and-miss combinations stored in the Integrated-Onset-Matrix (IOM) (Figure 1). We describe the process of compiling these matrices in the next section. If a six-pentad window matches a combination in the IOM, the corresponding earliest JP in the window is stored in a first-guess vector. For example, in (2.1) the binary combination between JP 30-35 (i.e., 1 1 1 0 1 1) matches one of the combinations in the IOM. JP 30 is then stored in the first-guess vector. Thus, the binary vector (2.1) yields the following first-guess vector of JP's matching IOM combinations:
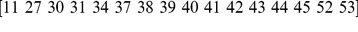
(2.2)


In contrast to other publications, where the authors have used a fixed cut-off date to avoid erroneously early onsets (e.g. May 10 in IMD forecasting procedure [25]), we analyze the time between all JP's in the first-guess vector (2.2) before we declare the onset date. We discard an entry as a false onset if the difference between two adjacent entries is more than nine pentads (45 days). Hence, the algorithm rejects JP 11 as the monsoon onset, because JP 11 occurs 16 pentads (80 days) before JP 27 in (2.2). The next two entries, JP 27 and JP30, are only three pentads (15 days) apart. Thus, JP27 is declared as the monsoon onset. We choose a separation criterion of nine pentads, as it accounts for the chance of break periods occurring shortly after the monsoon or rainy season onset [26].

The process for the withdrawal identification is exactly the same except we analyze in reverse order, from the end of the year (from JP 73) and backwards (towards JP 1) using the Integrated-Withdrawal-Matrix (IWM).

### 3. Compiling the Integrated Matrices

Compiling the Integrated-Onset-Matrix (IOM) and the Integrated-Withdrawal-Matrix (IWM) is an essential part of the IA process, where we integrate our understanding, knowledge and the requirements of the end-users or local communities. We justify this partly subjective input with a better feeling for the data and with the fact that manual interventions are still the cornerstone for some of the most well-known monsoon onset studies [12] and forecasts. The Indian Meteorological Department still declares the monsoon onset at Kerala when the duty forecasters decide that it has begun “*using a mixture of quantitative and qualitative methodologies*” [27], [28].

**Figure 2:**
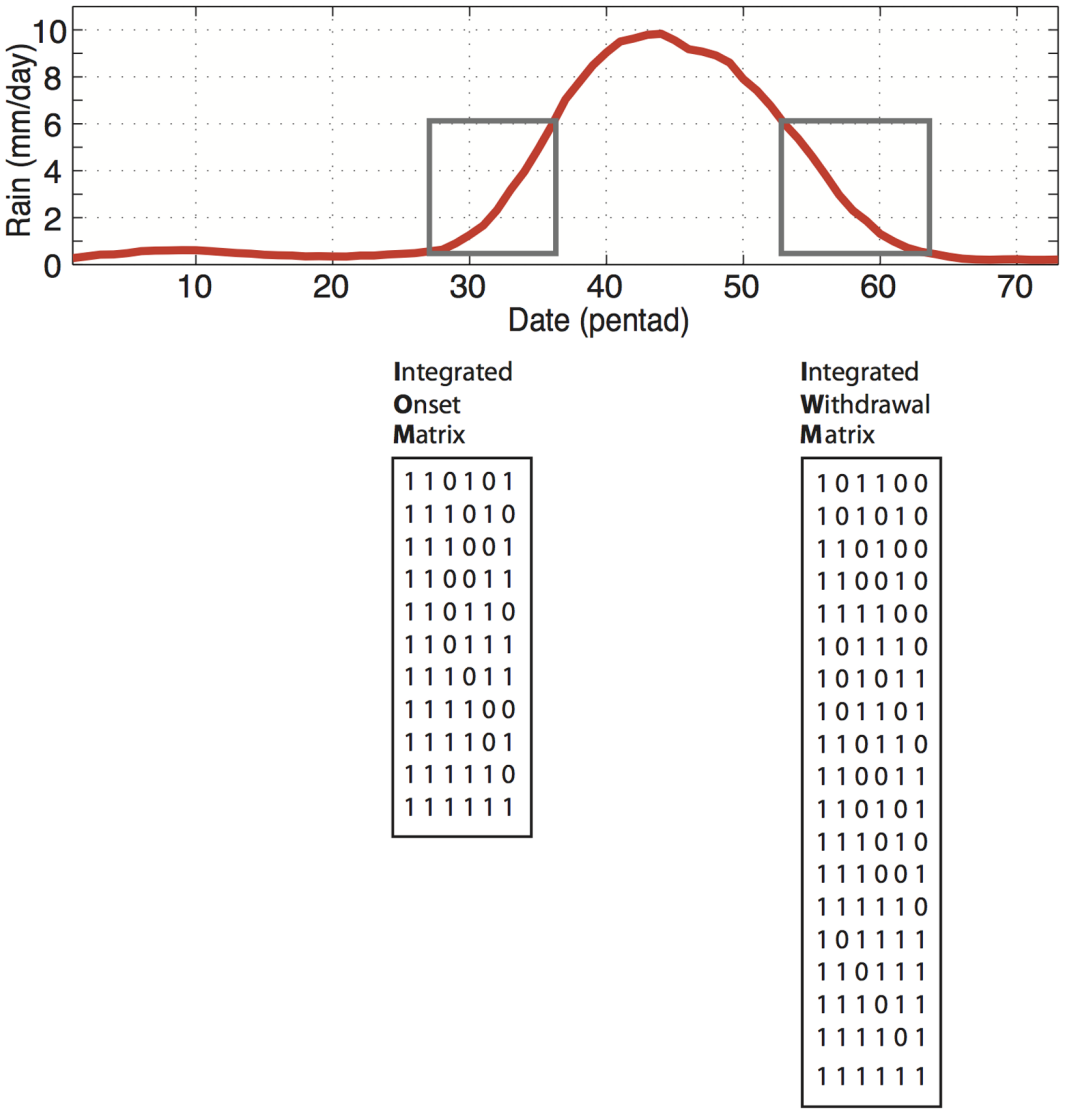
The figure shows that average rainfall (mm/day) for an area 80–90 E 22–25 N, with a 9-pentad running mean smoothing to illustrate the seasonal transitions. The figure shows that the increase in rainfall at the beginning is more rapid than the decrease at the end of the monsoon/rainy season. The slower transition is reflected in the Integrated-Withdrawal-Matrix (IWM), which contains more combinations than the Integrated-Onset-Matrix (IOM). The IOM and IWM contain all combinations of binary numbers used in the automatic identification process.

We compiled the integrated matrices by randomly choosing hundred grid points and years within the region of interest from the APRHODITE data set (1978–2007). After applying the definition to obtain the binary vectors, we manually allocated the onset and withdrawal pentad based on our prior knowledge. The algorithm then stores the 6-pentad combination starting at the chosen onset pentad and adds these combinations to the IOM. As the onset transition is more rapid compared to the withdrawal [15], [29], the IWM contains more combinations than the IOM. For example the combination [1 1 1 1 1 1] indicates a very sudden onset and is contained in both the IOM and the IWM. However, the combination [1 0 1 0 1 0] is a more gradual transition and is only contained in the IWM. The difference in transition speed is also evident in Figure 2, which shows the average 9-pentad running mean rainfall (mm/day) for a region in northern India. During the onset, the rainfall increases from 0.5 mm/day to 6 mm/day, faster than it decreases during the withdrawal.

Thus, the compilation procedure is more elaborate than other studies, but it allows us to adapt to the needs of the end-users and to incorporate further understanding of the monsoon climate. This process can be adjusted to different monsoon regions, where the respective local knowledge and meteorology can be included in the matrix compilation.

### 4. Test case set-up: domain, data and definition

To demonstrate the IA method, we focus on the region stretching from Bangladesh and westward across Northern India. Our original focus region was Bangladesh, but we wanted to test the IA method over a larger region. We extended the domain westward whilst keeping within Wang and LinHo's (2002) Indian Summer Monsoon region. Once we decided on the domain we needed to choose a definition and a data set to apply it to. In this test case, we apply the definition previously used by Matsumoto [15] on a high-resolution rainfall data.

We use the APHRODITE data set version V1101, which supplies daily precipitation values at 0.25° resolution over land between 1951–2007 [6], [7], [30]. Following Matsumoto's [15] set-up, the threshold chosen for this study is the multi-year (in our case, 1978–2007) mean pentad precipitation, which is a static value for each grid point. We apply our method to a region covering northern India and Bangladesh. This corresponds to the northern part of the Indian Summer Monsoon region as specified by Wang and LinHo [14].

While many use rainfall amounts as a proxy to identify the monsoon onset, Matsumoto [15] clearly states that his method identifies the summer rainy season. In order to be true to the original method, we emphasize that we identify the rainy season. Even though rainfall is a good proxy for the monsoon, there is not always a direct correspondence between the two concepts ([27] p133).

In the following, we compare two techniques for identifying seasonal transitions and verify the results against a purely manually corrected time series. The two techniques are the conventional three-pentad method, where three consecutive pentad ‘hits’ define the onset [15], [22], and the IA method. As there are over 2000 grid points in the chosen domain, it is not feasible to verify the techniques for all grid points in the entire domain.

If the IA reduces the occurrence of false onsets then this will, in principle, also reduce an erroneous spread in interannual variability. We therefore compare the standard deviations of the monsoon lengths for the entire domain for the results obtained with the IA and conventional method.

The main point of the test case is to investigate whether the 6-pentad IA method can reduce the number of false onsets compared to a more conventional approach. It is important to note that this is a demonstration and that different parameters, thresholds, and data sets might be more appropriate for other applications.

## Results and Discussion

We will assess how well the IA method performed at a single grid point and also over a wider region. We will start by looking at the time series from a single grid point and how the IA method compares with the conventional method. We benchmark the results against a purely manually corrected time series. Manual corrections have been widely used previously and are an intricate part of the IMD's ‘reanalysis’ monsoon onset time series [28].

The time series in Figure 2a illustrates a general association between the IA (red line) and conventional (black line) methods. The IA results precisely match the manually corrected benchmark time series (blue dots). This shows that the IA has clearly managed to eliminate false onsets and withdrawals. However the results in Figure 2a are from a single grid point and maybe not so convincing. We extended the test by manually identifying the onset and withdrawal at a further 100 random grid points and comparing to the IA and conventional method results. The IA method matched the manually identified onsets and withdrawals with 100% and 99%, respectively, whereas the conventional method matched 67% and 52%, respectively.

The IA and conventional methods give different onsets and withdrawals for several years. It is clear that we not only have a problem with false onsets, but false *withdrawals* also. This becomes evident when we compare individual years. Figure 2b shows that in 1979, both the IA and conventional methods disagree on the onset and withdrawal. The conventional method indicates a late onset and considerably early withdrawal, resulting in an unrealistic rainy season length of just four pentads. For 1986, both methods agree on the onset. However, three consecutive wet pentads late in the year resulted in the conventional method giving an erroneously late withdrawal (JP 73). The conventional method allocates a false onset in 1990. Three wet pentads from JP 25 caused the conventional method to declare this as the rainy season onset whereas the IA method gave an onset in JP 34. In 2000, the IA method was able to allocate the onset at the beginning of a reasonably wet period beginning JP 31, whereas the conventional method failed to capture this, and yielded an onset eight pentads later.

The differences shown in Figure 2 clearly influence calculations of interannual variability. Table 1 shows that the IA method (for the same grid point) gives standard deviations encouragingly similar to previous publications [12], [29], [30], [31]. The IA method gives an earlier average onset and a later average withdrawal. This translates to a 2-pentad longer rainy season when compared with results from the conventional method.

**Table 1:** Statistical values for the onset and withdrawal time series (1978–2007) for the grid point at 23.875 N, 83.875 E.

	Mean onset	Mean withdrawal	Mean length	Standard deviation onset	Standard deviation withdrawal
Conventional method	35.7	52.3	16.6	3.5	4.9
IA method	35.1	53.8	18.7	2.4	2.0

These results show that false onsets and withdrawals can swing both ways, and give erroneously early and late results. Consequently, the conventional method gives higher values of interannual variability.

The IA method seems to work at a selected single grid point, but how about over a larger region? Even if we have designed this method to be applied at local levels, we still want to know how the same monsoon definition performs over larger regions. Only then can we discuss the seasonal transitions in relation to large-scale processes and circulations.

We start our overview of the larger region by taking a look at the average onset and withdrawals for the IA and conventional methods. Then we will take a look at whether or not the IA has managed to reduce false onsets and withdrawals.

**Figure 3:**
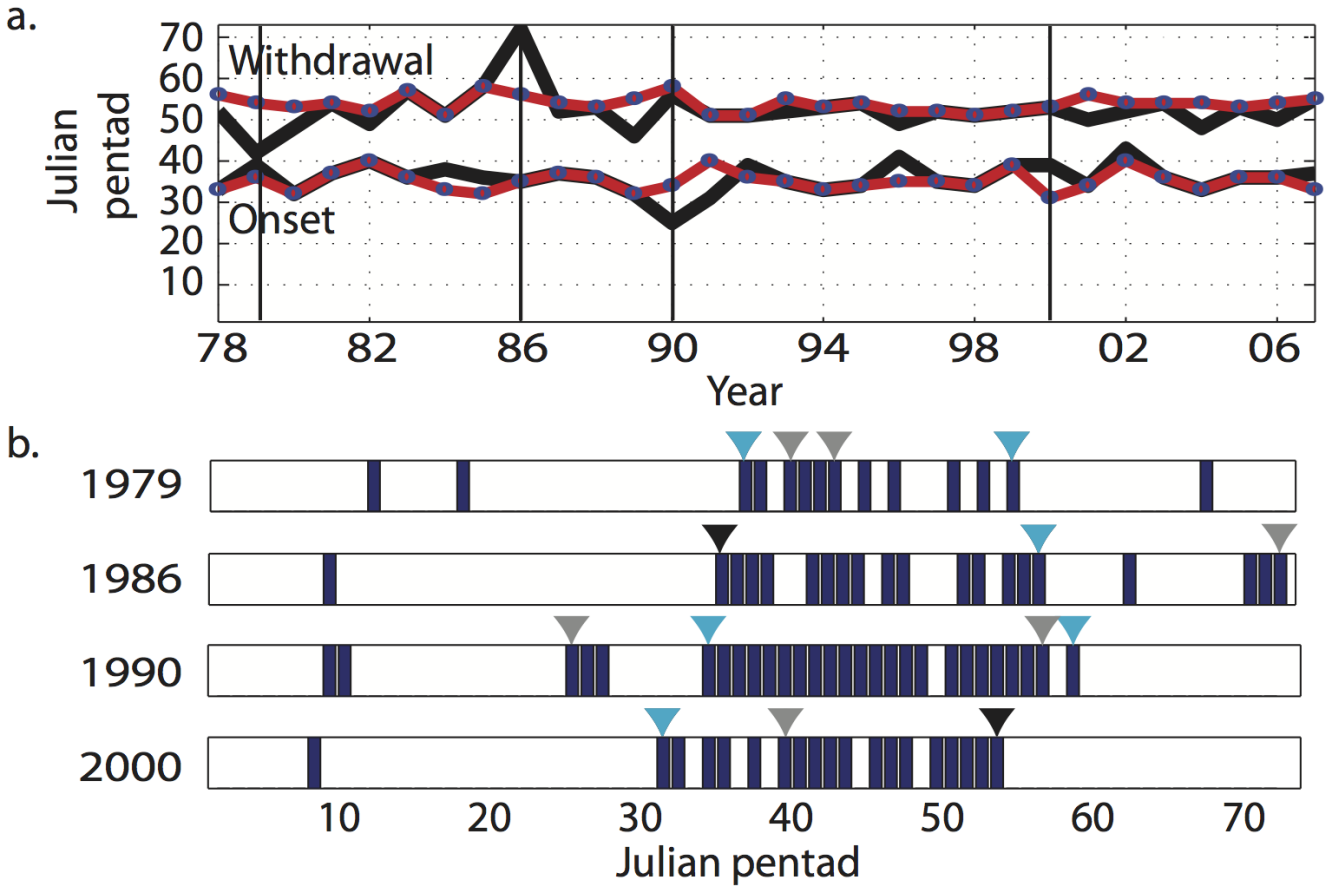
Comparison of the Integrated Approach (IA) and 3-pentad (conventional) methods (Matsumoto, 1997) for 23.875 N, 83.875 E. (a) The red line shows the IOM/IWM onset and withdrawal pentads for 1978–2007, where as the black line shows the results from the 3-pentad method. The blue dots show the purely manual corrected time series. (b) The blue bars show pentads where the threshold is exceeded. The grey arrows show onset/withdrawal pentad according to conventional method and the blue arrows show the IA method. Black arrows represent coincident onset/withdrawal pentads between the two methods.

From the multi-year averages, we can determine the onset progression and withdrawal regression over the whole region of interest. The onset progression looks similar when comparing results from the two methods (Figure 3) with a general direction from east to west, and a more north-easterly direction over North India. However, the IA method yields an earlier onset (roughly 1–2 pentads) over much of the region.

We observe particularly early onsets (around JP 25∼May 1) over northern Bangladesh and further north into the Brahmaputra Valley using both methods. This departs from most other monsoon onset climatological analyses [13], [16], [32], [33]. Despite others calling such early onsets ‘spatial discontinuities’ [13], we feel that these may illustrate subtle nuances in the monsoon or rainy season progression, that may have important implications for local community vulnerability.

**Figure 4:**
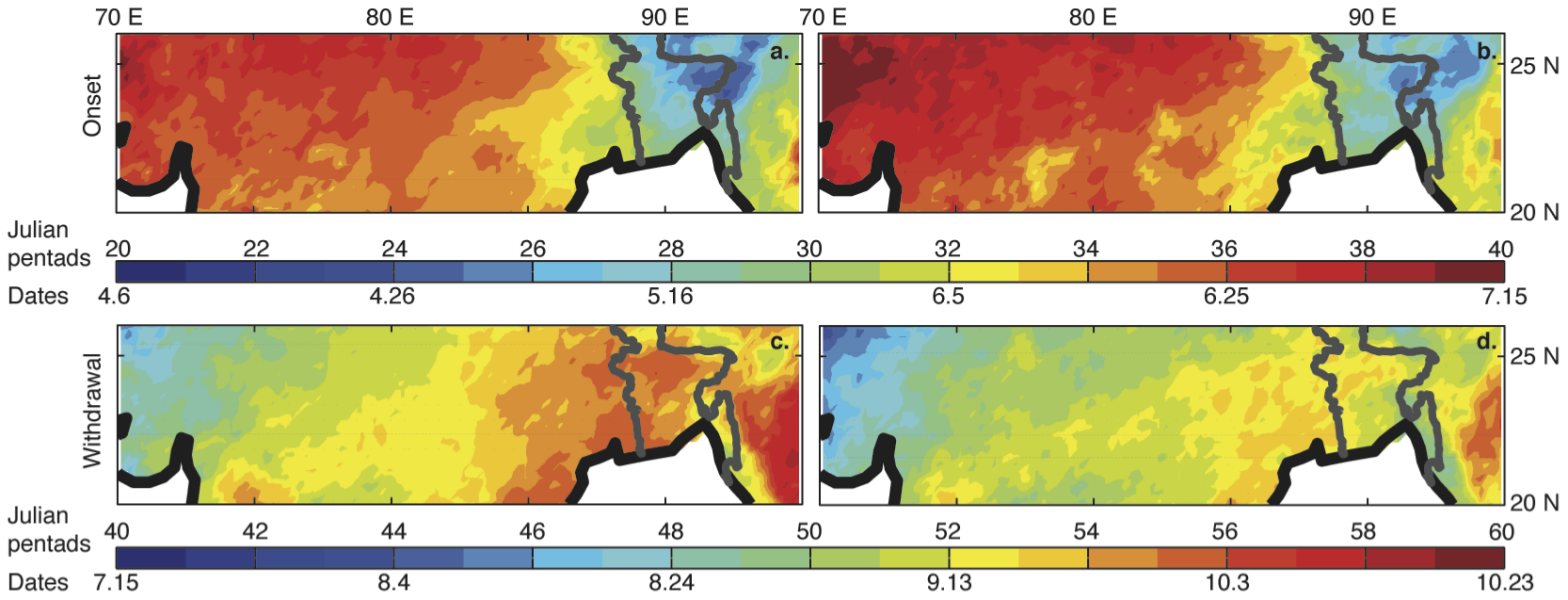
Multi-year mean onset pentads for (a) the Integrated Approach (IA) method and (b) the 3-pentad (conventional) method, across Northern India and Bangladesh. Also shown are the multi-year mean withdrawal pentads for (c) the Integrated Approach (IA) method and (d) the 3-pentad (conventional) method.

The average withdrawal patterns from the two methods are also similar (Fig. 4), but the IA method features a later withdrawal over much of North India and Bangladesh, with a delay of about 1–2 pentads. Generally, the IA method gives a slightly earlier onset and later withdrawal, with obvious implications on season length.

We want to examine if the IA manages to reduce false onsets and withdrawals. We cannot repeat the same grid point analysis as above because the whole domain contains over 2000 grid points. We have to find a different way to analyse the IA's effect. We discussed that if we reduce the occurrence of false onsets/withdrawals, then values of interannual variability would also decrease (shown by the decrease in standard deviation). We therefore look at the difference in standard deviation between the IA method results and the conventional method to indicate whether the IA reduces false onset/withdrawals.

**Figure 5:**
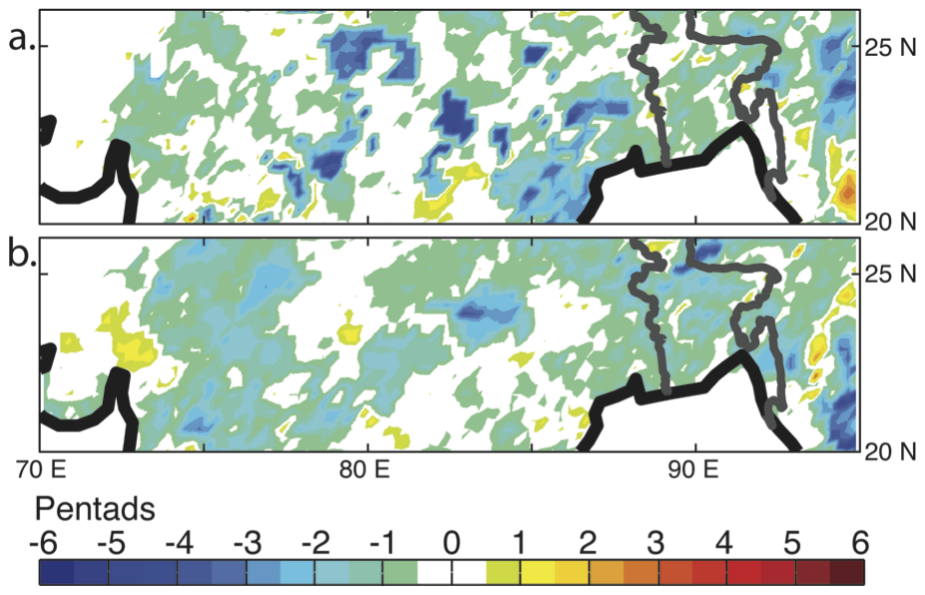
Differences in multi-year standard deviation between the IA and conventional methods for a). onset and b). withdrawal.

The IA method reduces the standard deviation of onset and withdrawal by up to 5 pentads in some regions (Figure 5a). For the onset, large differences in standard deviation are observed over the Pradesh region of northeast India (Figure 5a). For the withdrawal, consistently lower standard deviations are shown over most of Bangladesh for example (Figure 5b). Few regions show increases in interannual variability with the application of the IA methodology.

From the test case, we can see that the problem of false early onsets is extended to withdrawals and can be both erroneously early and late. We have seen that the IA method seems to match a purely manually corrected time series of onsets and withdrawals. This means that during the test phase the IOM and IWM managed to collate all the pertinent combinations of *hits* and *misses*. Over a larger region the IA method predominantly decreases the values of interannual variability. This is a good indication that the IA method manages to reduce the affect of false onsets and withdrawals in the resulting grid point time series.

## Conclusions

We applied the Integrated Approach (IA) and a conventional method to identify the monsoon onset and withdrawal dates in a gridded data set. We used the APRHODITE rainfall data as the gridded data, and analyzed the data between 1978–2007 over northern India and Bangladesh. Yearly precipitation data for each grid point was converted to pentad values and we applied the 30-year pentad mean as a threshold. Then the algorithm identified the monsoon onsets and withdrawal according to the transitions that the IA and conventional methods stipulated. We compared the results from the two methods to see if the IA method managed to achieve our first objective to reduce false onsets.

First, we investigated a single grid point to see how the IA affected false onsets. The IA reduced false onsets, and thereby also the interannual variability to levels similar to previous research. The IA also reduced erroneous withdrawals considerably. Hence, the standard deviation of withdrawal dates decreased by almost 3 pentads for the grid point we investigated. We extended the analysis over a larger region, showing that the IA method managed to reduce false onset and erroneous withdrawals over much of northern India and Bangladesh.

Secondly, we wanted to develop the IA to be flexible so that it could be adapted to different monsoon definitions. Due to the multitude of monsoon definitions, it is important to apply one that is relevant to the requirements of different stakeholders or users, whether they be other scientists, policy-makers or subsistence farmers. Our test case here demonstrates how we can use the approach and adapt it. If we responsibly choose relevant parameters, indices and thresholds, we should be able to apply the IA method to any monsoon definition. However, the flexibility and reductions in false onsets came at a price, as we invested significantly more time in designing and adjusting the IA.

We increased the preparation time needed the IA by extending the length of the windows to 6 pentads. With 6-pentad windows we needed to manually choose all the 6-pentad combinations that signify a monsoon onset or withdrawal. To do this responsibly, we applied prior knowledge and a manual testing phase. In our case, we knew that the monsoon onset starts more abruptly than the withdrawal. This knowledge influenced which 6-pentad transitions we choose during the manual testing phase, which is what took time. However, the more time we used, the better we understood our data. This understanding was not based on large-scale averages, or smoothed data. It was grounded in the raw grid point data. The IA basically forces us to consider the data at the local scale. This is valuable knowledge, especially if we wish to communicate with local communities, which is often the aim of project such as community-based climate adaptation.

However, if we want to communicate science responsibly to a local community, we should consider several issues. For example, if the local people of Bariyarpur and Maniknagar have to be informed about the monsoon onset, we pinpointed that reliable higher resolution data can be beneficial. To reap these benefits we must be able to apply these different definitions to our data. The fact that the IA approach reduces some problems when using high-resolution data and that it can be set-up according to different definitions means that it can ingest the opinions or requirements of a variety of stakeholders.

We will continue to work with stakeholders in northeast Bangladesh in a new international collaboration with researchers from Norway, Bangladesh and USA (including all the authors). Our main motivation is to provide climate information that is indeed relevant to and useable by the local agricultural communicates in northeast Bangladesh. The work we present in this paper is a step towards providing climate information about the monsoon that is relevant to its intended users.
